# Comprehensive haematological indices reference intervals for a healthy Omani population: First comprehensive study in Gulf Cooperation Council (GCC) and Middle Eastern countries based on age, gender and ABO blood group comparison

**DOI:** 10.1371/journal.pone.0194497

**Published:** 2018-04-05

**Authors:** Adhra Al-Mawali, Avinash Daniel Pinto, Raiya Al-Busaidi, Rabab H. Al-Lawati, Magdi Morsi

**Affiliations:** 1 Centre of Studies & Research, Ministry of Health, Muscat, Sultanate of Oman; 2 The Royal Hospital, Muscat, Sultanate of Oman; Zentralkrankenhaus Bremen Mitte, GERMANY

## Abstract

**Background:**

Reference intervals for venous blood parameters differs with age, gender, geographic region, and ethnic groups. Hence local laboratory reference intervals are important to improve the diagnostic accuracy of health assessments and diseases. However, there have been no comprehensive published reference intervals established in Oman, the Gulf Cooperation Council or Middle Eastern countries. Hence, the aim of this study was to establish reference intervals for full blood count in healthy Omani adults.

**Methods:**

Venous blood specimens were collected from 2202 healthy individuals aged 18 to 69 years from January 2012 to April 2017, and analysed by Sysmex XS-1000i and Cell-Dyn Sapphire automated haematology analysers. Results were statistically analysed and compared by gender, age, and ABO blood group. The lower and upper reference limits of the haematology reference intervals were established at the 2.5th and 97.5th percentiles respectively.

**Results:**

Reference intervals were calculated for 17 haematology parameters which included red blood cell, white blood cell, and platelet parameters. Red blood cell (RBC), haemoglobin (HGB), haematocrit (HCT), platelet and platelet haematocrit counts of the healthy donors were significantly different between males and females at all ages (p < 0.05), with males having higher mean values of RBC, HGB and HCT than females. Other complete blood count parameters showed no significant differences between genders, age groups, instruments, or blood groups. Our study showed a lower haemoglobin limit for the normal reference interval in males and females than the currently used in Oman.

**Conclusions:**

Data from this study established specific reference intervals which could be considered for general use in Oman. The differences in haematology reference intervals highlights the necessity to establish reference intervals for venous blood parameters among the healthy population in each country or at least in each region.

## Introduction

It is hard to underestimate the importance of clinical laboratory test results. Nearly 80% of physicians' medical decisions are based on information provided by laboratory reports [1]. Test results by themselves are of little value unless it is reported with the appropriate information for its interpretation. Typically, this information is provided in the form of a reference interval [2, 3].

Full blood count (FBC), also known as complete blood count (CBC), testing is the most frequently performed haematology test in any clinical setting. Analysis of FBC by haematology analysers is an essential and basic tool in the evaluation of many immunological, haematological and pathological disorders. Establishment of a normal reference interval is fundamental for precise clarification of disease diagnosis based on age, gender, ethnicity, genetic differences, and environmental factors [4].

FBC techniques have developed considerably in the last two decades and automated methods have now replaced manual methods. Automated haematology analysers utilise technological advancements to determine complete blood count more accurately. In addition, various novel blood cell parameters have been developed alongside to aid in diagnosis and management of several blood disorders [5–8].

Demographic, environmental, geographical area, and ethnic population are factors that may contribute to variations in these ranges around the world [9–11]. Dietary habits and occupational exposures are also factors that has been shown to affect reference interval [12–14]. Country-specific reference intervals for FBC of adult peripheral blood have been established in many countries around the world; however, there have been no specific comprehensive studies in Oman or the Gulf Cooperation Council (GCC) and Middle East, which investigated reference interval based on age, gender-specific, and ABO blood group. Due to the variability observed as a result of the factors, there is a great necessity for each country to establish its own reference intervals.

In Oman, there is no single study has been done to identify the reference interval for FBC. To the best of our knowledge, this is the first study in Oman and GCC that investigated reference interval; and the data was correlated with age, gender, and blood group, and two haematology analysers. This study identified reference interval for all FBC parameters, which can now be used as a reference when evaluating patient samples in Oman.

## Materials and methods

### Subjects and sample collection

The Central Blood Bank (CBB) is the primary blood bank for Ministry of Health in the Muscat region as well as referred samples from all over Oman. As per standard blood donation procedure, a blood donation form has to be filled prior to determining eligibility. Selection criteria for blood donation are based on a questionnaire which is given to each volunteer donor to assess their medical, demographic, and lifestyle information in addition to a physical examination by a clinician to determine fitness to donate blood. The exclusion criteria for blood donation is as below:

- Age below 18 years;
- Any type of illness, such as cancer, acute or chronic infection, diabetes, allergy or asthma requiring long-term treatment, epilepsy, cardiac or respiratory disease;
- Surgery in the past 12 months prior to donation;
- Blood donation within the past 3 months;
- Body weight under 50kg;
- Hypertension;
- Any bleeding condition/blood disorder requiring medical attention
- Vaccination taken in the past 4 weeks;
- Diarrhoea in the past 2 weeks;
- Pregnancy, or abortion/delivery within the past 12 months
- Tattoos, ear or skin piercings, acupuncture, or traditional ‘*wasam*’ (local cautery) treatment/*hijama* (cupping therapy) in the past 12 months;
- HIV/AIDS, Hepatitis, Syphilis, or a sexually transmitted disease;
- Travel to malaria endemic areas, or development of febrile illness upon return

Informed consent which is taken as part of the blood donation process also covers the haematological parameters tested in this study. Ethical approval for the study was obtained from the Research and Ethical Review and Approval Committee, Ministry of Health. During blood donation, peripheral blood specimens were also collected in 2ml K3EDTA vacuette tubes by venepuncture for the reference interval study. A convenient sample of 2202 eligible apparently healthy normal Omani adult blood donors from CBB were taken for this study during the period of January 2012 to April 2017. Of the 2202 volunteer healthy eligible blood donors, 1879 were males and 323 were females. The blood donors were between 18 and 69 years of age (median 30 years). The blood specimens were collected in the morning (8am-12pm) and processed in the laboratory within 5 hours of blood collection.

### Measuring instruments and parameters

The collected blood samples were analysed at the Royal Hospital (largest tertiary hospital) in the Sultanate of Oman. FBC parameters were measured using Sysmex XS-1000i (Sysmex Corp., Kobe, Japan) and Cell-Dyn Sapphire (Abbott Laboratories, Diagnostic Division, Abbott Park, IL, USA) haematology analysers. Currently, 95% of laboratories in Oman apply these two analysers in their routine practice. 17 haematology parameters were evaluated in this study which comprised red blood cell parameters of red blood cells count (RBC), haemoglobin (HGB), haematocrit (HCT), mean corpuscular volume (MCV), mean corpuscular haemoglobin (MCH), mean corpuscular haemoglobin concentration (MCHC), red cell distribution width (RDW); white blood cell parameters of white blood cells count (WBC), absolute neutrophil count (NEUT), absolute lymphocyte count (LYMPH), absolute monocyte count (MONO), absolute eosinophil count (EOS) and absolute basophil count (BASO); and platelet parameters of thrombocyte/platelet count (PLT), platelet distribution width (PDW), mean platelet volume (MPV), and platelet crit (PCT). Both instruments were evaluated and a correlation between instruments was performed.

### Quality control & Quality assurance

Quality controls (3 different levels of low, medium and high) for the respective instruments provided by Abbott, USA and Sysmex, Japan were run daily as part of the internal quality control thrice a day. Calibration of instruments as well as quality control monitoring between instruments was carried out regularly as part of scheduled maintenance to ensure precision and reliability of results. The laboratory is covered by UKAS Medical Laboratory accreditation (ISO 15189).

### Statistical analysis

**Fig 1:**
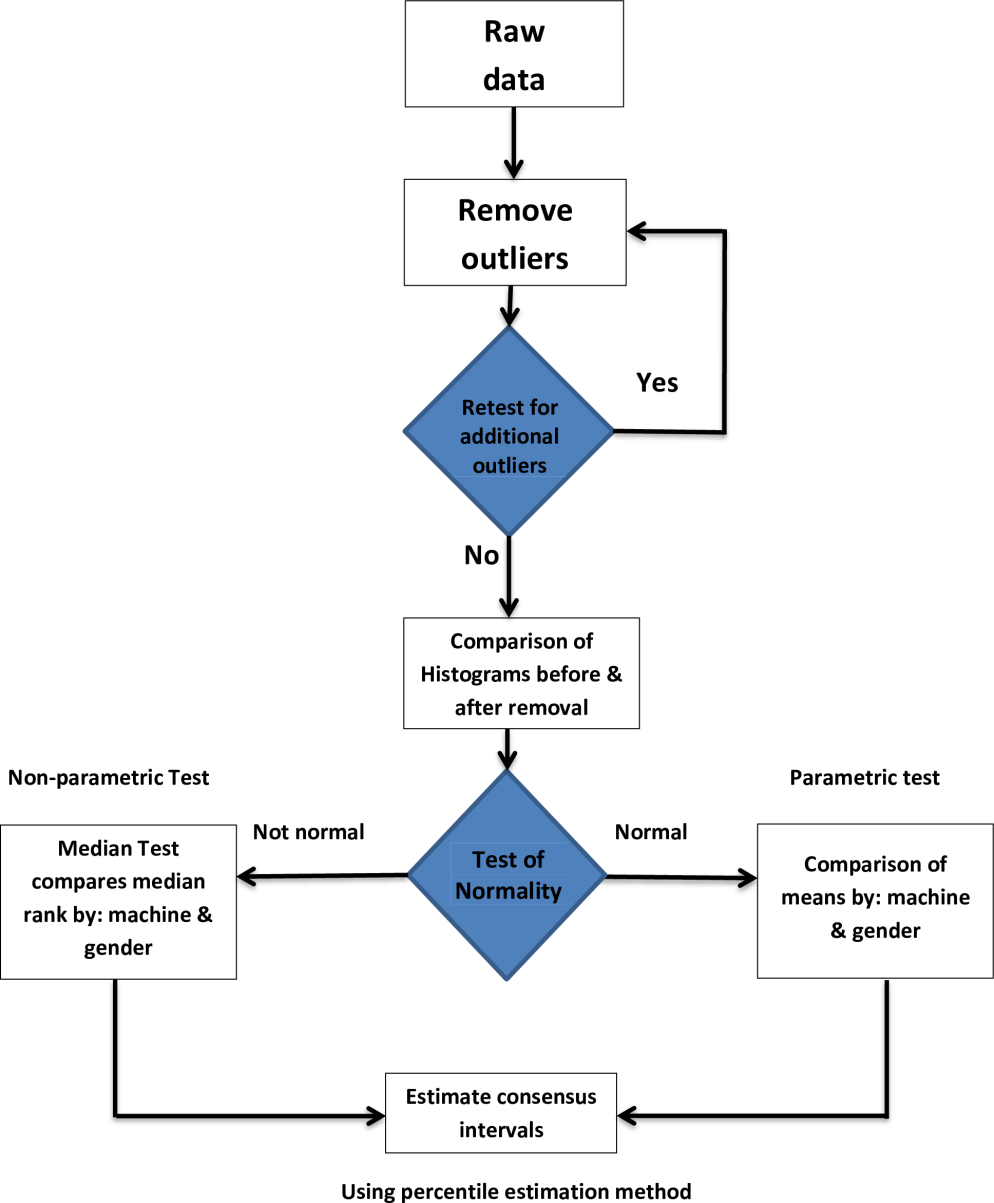
Statistical methodology employed in estimating reference intervals. Lower and upper reference limits were determined using the 2.5th and 97.5th percentiles.

Data were analysed using the IBM Statistical Package for Social Sciences (SPSS Version 20.0). Outliers were excluded by Horn’s algorithm using Tukey’s interquartile fences identifies multiple outliers located at the upper and lower extremities [15]. The criterion for rejection is values exceeding interquartile (IQ) fences set at Q_1_-1.5*IQR and Q_3_+1.5*IQR (where IQR = interquartile range; IQR = Q_3_-Q_1_ where Q_1_ AND Q_3_ are the 25^th^ and 75^th^ percentiles, respectively. This is more stringent than Dixon’s range statistic, which favours retention. The two instruments (Sysmex and Cell-Dyn) and the two genders were compared using the Kolmogorov-Smirnov Z-test and the Median test whilst comparisons in age groups were done using ANOVA and Kruskal-Wallis H test. For multiple comparisons of groups that showed differences in variances analysis, Tukey’s test was used. Non-parametric methods were used to establish the FBC values between the 2.5^th^ and 97.5^th^ percentiles that included 95% of the reference sample group data (Fig 1).

## Results

### Instrument-related findings

**Fig 2:**
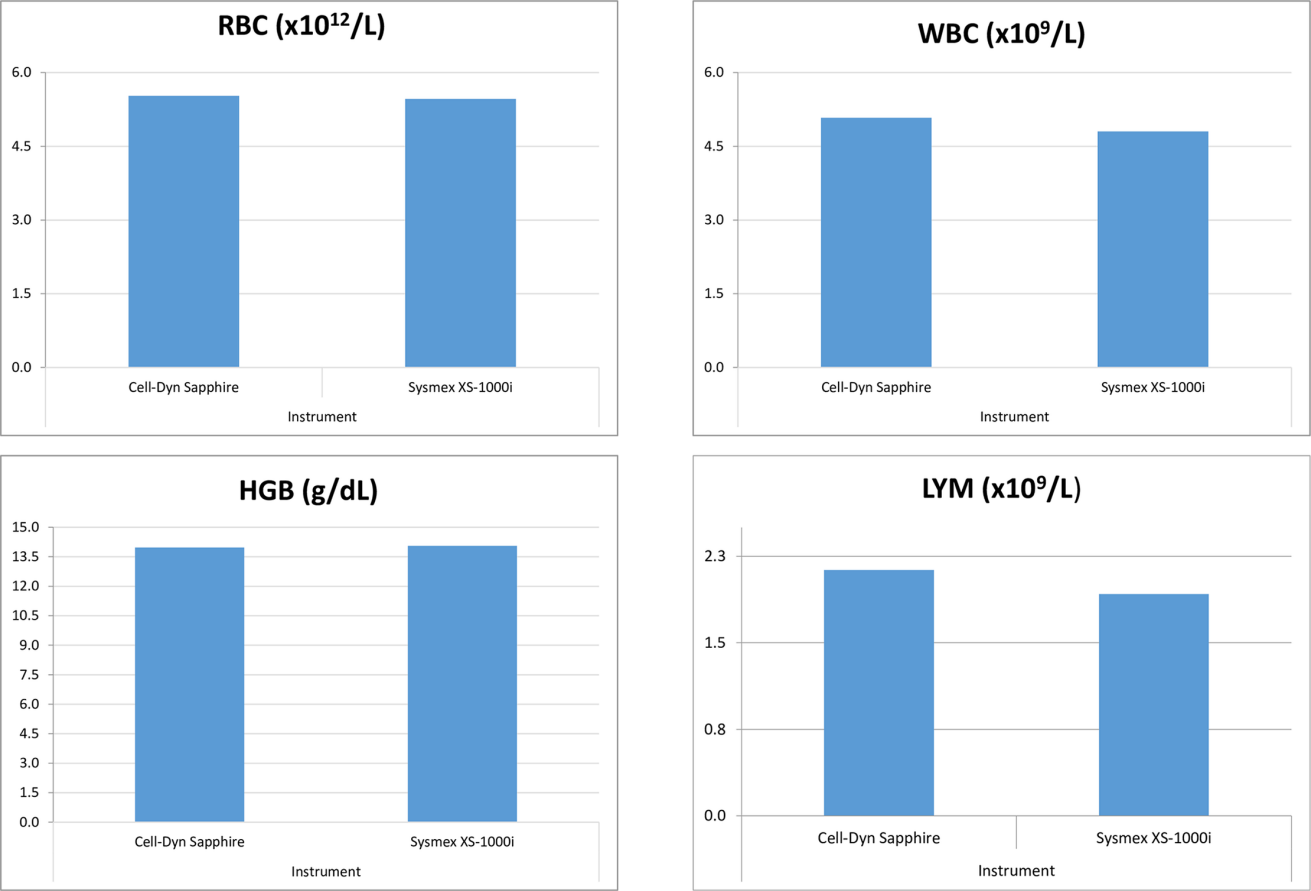
Comparison of mean red blood cell count (RBC), white blood cell count (WBC), haemoglobin (HGB), and lymphocyte (LYM) values between different instruments. Instrument 1 denotes Cell-Dyn Sapphire and Instrument 2 denotes Sysmex XS-100i.

Statistically significant differences were seen only in PLT and LYMPH using the two instruments. However, the differences between the mean values were minor and not clinically significant, and thus the data from both instruments were pooled together to calculate the reference intervals for the total population (Fig 2).

### Gender-related findings

**Fig 3:**
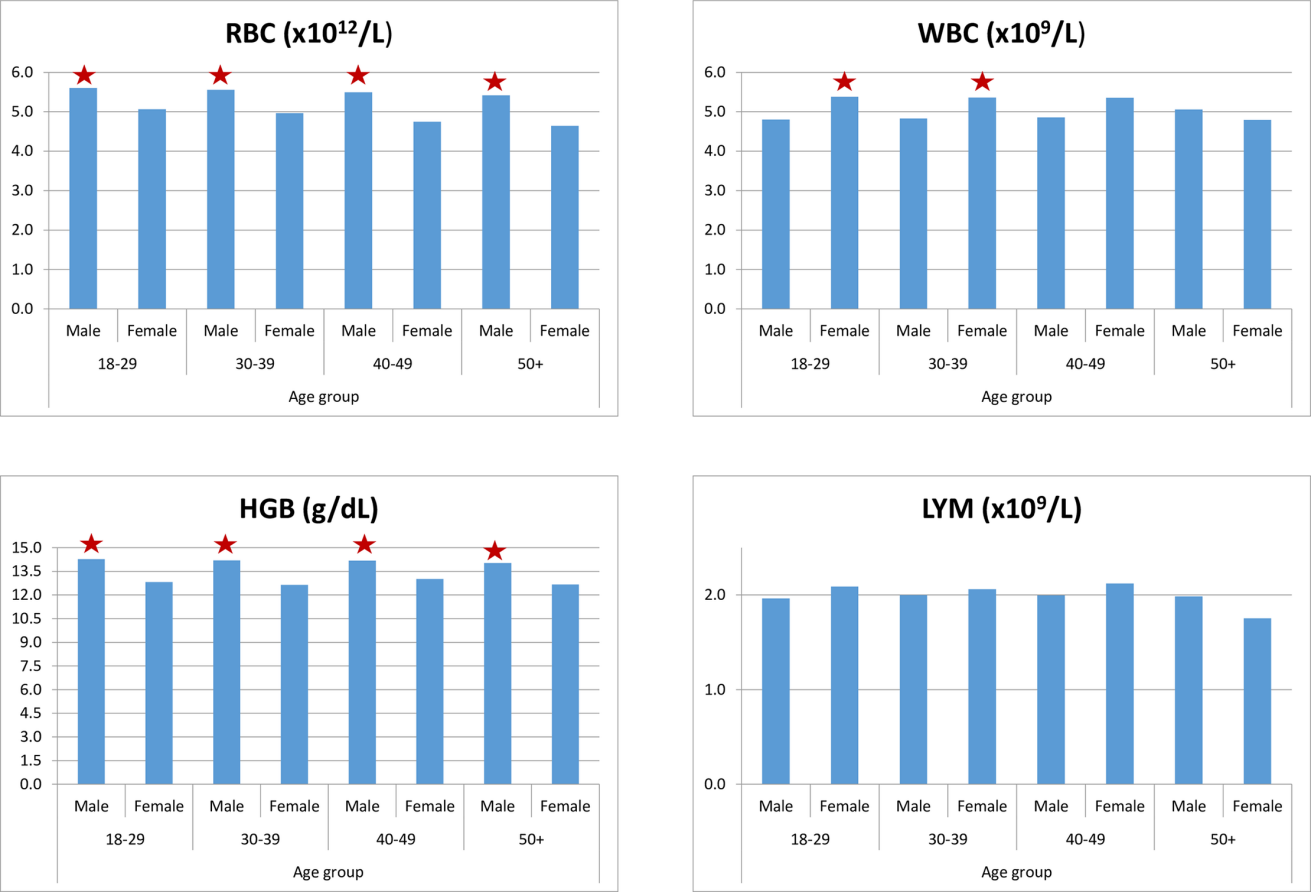
Variations of mean red blood cell count (RBC), white blood cell count (WBC), haemoglobin (HGB), and lymphocyte (LYM) values according to age and gender. * denotes significantly higher values (P < 0.05) between genders in the same age group.

Significant differences were found between males and females (P < 0.05) for RBC, HGB, HCT, PLT parameters (Fig 3). The rest of the parameters showed no statistical differences between males and females and thus the data was pooled together whilst preparing the reference intervals.

### Age-related findings

The findings were divided into 4 age groups: 18–29, 30–39, 40–49, and 50+. Very minor differences with no statistical significance were seen among the different age groups and hence the data between age groups were pooled in together (Fig 3).

### Blood group-related findings

**Fig 4:**
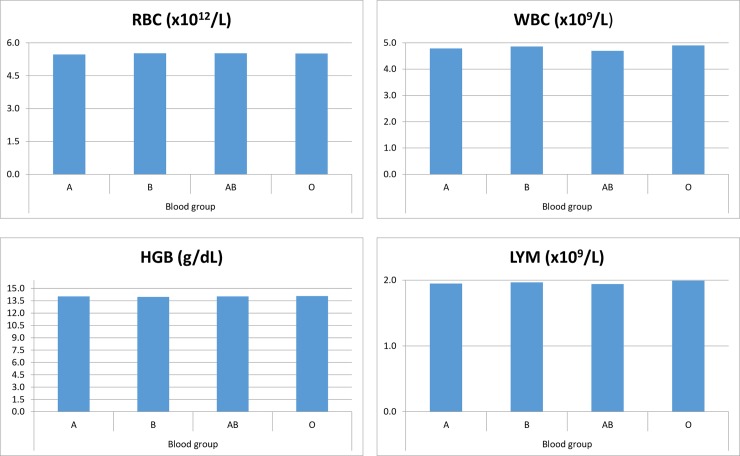
Comparison of mean red blood cell count (RBC), white blood cell count (WBC), haemoglobin (HGB), and lymphocyte (LYM) values amongst ABO blood group. The selected analytes show no significant difference amongst ABO blood group.

There were no statistical differences found in the subjects between the four blood groups of A, B, AB & O and their respective Rh factors (Fig 4).

### Estimating blood reference intervals

The reference intervals did not differ by gender, except for RBC, HGB, HCT, and PLT. Lower and upper reference limits were determined using the 2.5^th^ and 97.5^th^ percentiles, shown in Table 1. The qualified rate of validation which is the percentage of donors falling in the established reference interval was not less than 95% for all parameters (based on CLSI guideline for verification of reference intervals) [16].

**Table 1:** Estimated reference intervals of different FBC parameters.

Analytes	Sex	Reference Interval	Qualified rate of validation
RBC (x10^12^/L)	M	4.45–6.75	95.1%
	F	4.07–6.17	95.5%
HGB (g/dL)	M	12.4–16.4	95.8%
	F	11–15.1	96.5%
HCT (L/L)	M	0.36–0.47	95.3%
	F	0.33–0.43	95.5%
PLT (x10^9^/L)	M	146–347	95.0%
	F	164–368	95.3%
PCT (%)	M	0.13–0.28	96.6%
	F	0.15–0.30	98.9%
MPV (fL)	M/F	6.61–10.3	95.3%
MCV (fL)	M/F	62.5–88.5	95.1%
MCH (pg)	M/F	20.81–31.2	95.1%
MCHC (g/L)	M/F	31–37.2	95.6%
RDW (%)	M/F	11.1–17.8	95.4%
PDW (%)	M/F	7.7–17.4	95.2%
WBC (x10^9^/L)	M/F	2.79–8.09	95.2%
NEUT (x10^9^/L)	M/F	0.91–4.61	95.0%
LYMPH (x10^9^/L)	M/F	1.12–3.15	95.2%
MONO (x10^9^/L)	M/F	0.23–0.72	95.8%
EOS (x10^9^/L)	M/F	0.03–0.37	95.1%
BASO (x10^9^/L)	M/F	0.002–0.058	95.2%

## Discussion

The FBC is the most commonly requested diagnostic haematology laboratory test. A reference interval set up for FBC aids clinicians in diagnosis and evaluation by distinguishing between a normal and a pathological situation. Technological advances in automation contribute to the need to set up and consequently periodically evaluate reference intervals in order to ensure the requirements of quality and precision. Different laboratories are urged to define reference intervals for the local population as well as providing national recommendations based on the established reference intervals because FBC values are impacted by various factors, which include age, gender, altitude, ethnic background [17]. Thus it is important that each country or region establish their own reference interval for FBC parameters.

To the best of our knowledge, this is the first study that reports comprehensive reference intervals for the full range of diagnostic haematology parameters in Oman, GCC and the Middle East. This study investigated the influences of instrument type, age, gender, and blood group on the FBC reference intervals in healthy donors of the Omani population as they presented to the Central Blood Bank.

In our study, RBC reference intervals reported are similar to the reported reference intervals by American, Malaysian, and French studies [18–20]. The upper RBC limit for both males and females was higher in Oman (Table 2) which was similar to an African study [21]. This study is also in line with the general knowledge about the significant difference in haemoglobin levels between males and females as a result of the effects of androgen and oestrogen on erythropoiesis [22]. The HGB values are lower than the recommended reference intervals by World Health Organization (WHO) for non-anaemic persons with the severity in Oman being assessed as ‘Severe’ [23, 24]. The World Health Survey in Oman, a community-based household survey, reported the prevalence of anaemia to be 28% in the Omani population taking into consideration the WHO haemoglobin lower limits of 13g/dL and 12g/dL for males and females respectively [25], with prevalence of anaemia among non-pregnant women (aged 15–49) at 35% as of 2011 [26]. However, this study shows this is actually a normal reference interval for the Omani population. Interestingly, a similar finding was also seen in Kuwait, another Middle Eastern country [27]. Our lower HGB limit for females are similar to Malaysian & Chinese populations, whilst the lower HGB limit for males are similar to an African study [28]. Lower haemoglobin levels in our cohort may be attributed to the existence of genetic blood disorders [29, 30]. As predicted by the haemoglobin differences between males and females, a significant difference is similarly found in the haematocrit values between genders in our study which is similar to other published data [18, 19] due to higher androgen levels in males and chronic menstrual blood loss in females [31].

**Table 2:** Comparison of estimated reference intervals of different FBC parameters between various populations.

Analytes	Sex	Reference Interval	Current reference interval used in Oman ^(S^^1^ ^Ranges)^	American^[^^17^^]^*	Malaysian^[^^18^^]^^^^	African^[^^21^^]^*	Chinese^[^^31^^]^*
RBC (x10^12^/L)	M	4.45–6.75	4.5–5.8	4.5–5.9	4.53–5.95	4.0–6.4	4.28–5.81
	F	4.07–6.17	4.1–5.4	4.0–5.2	3.87–5.21	3.8–5.6	3.81–5.13
HGB (g/dL)	M	12.4–16.4	11.5–15.5	13.5–17.5	13.5–17.4	12.2–17.7	13.3–17.5
	F	11–15.1	11–14.5	12–16	11.6–15.1	9.5–15.8	11.5–15.2
HCT (L/L)	M	0.36–0.47	0.35–0.45	0.41–0.53	0.401–0.506	0.35–0.51	0.40–0.51
	F	0.33–0.43	0.34–0.43	0.36–0.46	0.351–0.449	0.29–0.45	0.35–0.46
PLT (x10^9^/L)	M	146–347	140–400	150–350	142–350	126–438	127–350
	F	164–368	150–450		171–399		
PCT (%)	M	0.13–0.28	-				-
	F	0.15–0.30					
MPV (fL)	M/F	6.61–10.3	M: 7.2–10.5 / F: 7–10.5				
MCV (fL)	M/F	62.5–88.5	M: 78–96 / F: 78–95	80–100	80.6–95.5	68–98	82.3–99.2
MCH (pg)	M/F	20.81–31.2	26–33	-	26.9–32.3	-	27.0–33.7
MCHC (g/L)	M/F	31–37.2	31–35	-	31.9–35.3	-	31.6–35.4
RDW (%)	M/F	11.1–17.8	11.5–16.5	-	12–14.8	-	-
PDW (%)	M/F	7.7–17.4					-
WBC (x10^9^/L)	M/F	2.79–8.09	M: 2.2–10 / F: 2.4–9.5	4.5–11.0	4.078–11.370	3.1–9.1	3.64–9.39
NEUT (x10^9^/L)	M/F	0.91–4.61	M: 1–5 / F:1–4.8	2.0–7.0	3.929–7.147	1.0–5.3	1.80–6.30
LYMPH (x10^9^/L)	M/F	1.12–3.15	M: 1.2–4 / F: 1.2–3.8	1.0–4.8	1.847–4.807	1.2–3.7	1.06–3.20
MONO (x10^9^/L)	M/F	0.23–0.72	M:0.2–0.6 / F: 0.2–0.5	0.12–1.00	0.385–1.141	0.2–0.78	0.16–0.62
EOS (x10^9^/L)	M/F	0.03–0.37	0.1–0.5	0.02–0.50	0.0–0.827	0.04–1.53	0.02–0.52
BASO (x10^9^/L)	M/F	0.002–0.058	0–0.2	0–1	0.0–0.095	0.01–0.15	0.00–0.06

*Community-based study recruitment

^ Voluntary participation recruitment

Both the upper and lower limits of MCV values are lower than those found in America, Malaysia,China, and Kuwait [17, 18, 27, 31]. Only an African study had its lower MCV limit similar to that found in our study [28]. A possible explanation to the low MCV values seen in our population is the high prevalence of thalassemia’s in the Omani population [32]. It is critical to segregate the population into two groups to establish a more accurate reference interval in the future as molecular analysis is not done in this study. The Clinical and Laboratory Standards Institute (CLSI) guidelines are unclear on how to proceed in the event of apparently healthy individuals having asymptomatic conditions impacting on reference intervals [16]. However, the reported MCHC values in our population is in line with other populations [18, 31]. Although donors are generally considered to be healthy people, they may suffer from iron depletion and subclinical iron deficiency which may have effects on certain red blood cell parameters.

PLT values showed significant differences between males and females in our population. The differences in platelet values between genders has been seen in other studies but the causes of the variation are unclear [33]. Our platelet values were similar to that found in the American and Malaysian studies [18, 19]. However, there is a noticeable difference when compared to African and Chinese studies which had a lower PLT limit [28, 31].

With respect to leukocytes, our population has much lower WBC and NEUT limits when compared to other populations, though the lower NEUT limit is similar to that reported in an African study population [28]. Our NEUT reference interval results is similar to the ranges previously reported by an Omani study on 126 donors [34]. Interestingly, ‘benign’ neutropenia is prevalent among Arabs [35, 36] and is possibly caused as a result of a genetic adaptation with its molecular mechanism still unknown [37]. This evidence and variations among different ethnic groups thus supports the necessity of establishing a local reference interval for Omani’s, so as to avoid any adverse consequences of wrong classification or misdiagnosis as neutropenia. However, the rest of the leukocyte parameters are in line with other international studies.

### Limitations

One of the limitations of this study is that blood donors are not the ideal population for healthy individuals but they are representative of ‘apparently’ healthy people. In addition, the number of women in the study were far less than men because the donors were volunteer blood donors coming for blood donation and there are more stringent criteria for women to donate according to the local guideline followed adapted from the WHO Blood Donor Selection Guideline [38]. Furthermore, even though a convenient sample has been taken, it is unlikely to have caused a selection bias due to the large sample size collected and the inclusion of consecutive eligible donors on the days of selection. CLSI guidelines recommend the establishment of reference intervals with at least 120 reference individuals using a non-parametric approach [16].

This study only included adults and not paediatric subjects due to eligibility criteria for donor blood collection. Another limitation is the number of 50+ individuals were considerably lower than the other age groups which may have had an impact on the reference intervals in this age category.

## Conclusion

This study successfully created a set of reference intervals for the majority of haematology parameters studied in healthy adult Omani population based on age, gender, and ABO blood group using two instruments. These reference intervals have important clinical implications, especially in the management of immunological and haematological disorders. The authors stress that each laboratory must validate the transference of established reference intervals (according to CLSI guideline). The reference interval established by this study could be adopted as a reference for clinical practice decisions in Oman. A follow-up study will need to be done to validate our results with iron status tests.

## Supporting information

S1 Ranges Current reference intervals used in Oman. (PDF)
