# Supplementary material for: Comprehensive haematological indices reference intervals for a healthy Omani population: First comprehensive study in Gulf Cooperation Council (GCC) and Middle Eastern countries based on age, gender and ABO blood group comparison
Source: PLoS One. 2018 Apr 5;13(4):e0194497. doi: 10.1371/journal.pone.0194497 (PMC5886408; doi:10.1371/journal.pone.0194497)
Supplement: S1 Ranges — (PDF) [file pone.0194497.s001.pdf]

1. **PURPOSE:**
   The purpose of complete blood count is used to evaluate the compositions and concentration of blood cells. Complete blood count provides 22 parameters related to blood cells. For example total white blood cell count with the neutrophil, lymphocyte, monocyte, eosinophil and basophil count. Red blood cell count with hemoglobin concentration, hematocrit, mean corpuscular volume, mean corpuscular hemoglobin concentration and red cell distribution width. Platelet count with mean platelet volume, platelet distribution width, and plateletcrit.

2. **PRINCIPLE**:
   The CD Sapphire counts, sizes and classifies red cells, white cells and platelets. It utilises three types of measurements to the task of blood cell analysis:-
   1. Optical flow cell measurement (MAPSS).
   2. Impedance transducer measurements.
   3. Haemoglobin flow cell measurements.

   Optical flow cell and impedance transducer measurements follow the principles of flow cytometry. Haemoglobin flow cell measurements are carried out using absorbance spectrophotometry.

3. **SAFETY:**
   Universal Precautions must be practiced at all stages of operation: HSM/OHSE/2

4. **PERSONNEL AND RESPONSIBILITY:**
   All technical staff in the Haematology laboratory trained to perform the test, interpret and then release the result. Haematology supervisor will check the report before releasing only if needed.
   Refer to form FM/HAEM/ROUT/04 Comprehension and competency staff list authorized to do the procedure.

5. **SAMPLE:**
   Blood collected by venepuncture into a purple top EDTA tube that will draw 2 ml. Samples must be mixed by inversion immediately after collection. Minimum volume of sample used for this test is 1ml. Test should be performed within 24 hours after collection. Sample will be stored at 4°C for 1 week.

The colored ink stamp indicates this is a controlled document. Absence of color indicates this copy is not controlled and will not receive revision updates.

6. MATERIALS:

Reagents	Consumables	Equipment
Patient EDTA tube	Printer Paper	CD Sapphire machine
Diluent Sheath reagent		Printer
WBC A & B		
Hb Reagent		
Tri-level controls		
CRM – Calibrators		

The stability of Diluent Sheath, WBC part A, part B and Hb reagent is up to expiry date at room temperature. Source of reagents is Abbott.

7. REAGENT PREPARATION:

Not applicable to this method, commercial prepared reagent available.

8. QUALITY CONTROL:

Internal QC: - Tri-level controls, Run every shift
Moving average program (X-B)
External QC: - UKNEQAS every month

9. PROCEDURE:

Refer to TP/HAEM/ROUT/13

9. CALCULATIONS:

Not required for this procedure.

10. LINEARITY:

Refer to TP/HAEM/GEN/12

11. INTERPRETATIONS / RESULTS / ALERTS:

The results are transmitted automatically to the host computer after the completion of the run and the reading of the tubes barcode. Interpretation of results is based on reference range, gender and age, at the same time the report should be compared with previous report (Delta check) along with scatter plots on the screen.
For critical values refer to QP/28 and FM/HAEM/ROUT/29

12. PROCEDURE NOTES:

CHECK FOR SAMPLE CLOTS BEFORE PERFORMING THE TEST.

The colored ink stamp indicates this is a controlled document. Absence of color indicates this copy is not controlled and will not receive revision updates.

13. METHOD PERFORMANCE SPECIFICATIONS:

Specificity and sensitivity of the Abbott CD Sapphire system has been stated to be 90-95%.

14. RELATED DOCUMENTS:

**Document Name**
Safe Work Practice PPC
Healthcare waste management
CD Sapphire Operation
Daily Internal QC-Record – Celldyn Sapphire
Cell Dyn Sapphire Daily Maintenance Schedule
Cell Dyn Log Sheet
Weekly &Manthly Maintenance schedule for Cell-dyn Sapphire
Method Validation
Critical/ Panic Values Policy
Critical Values Log

15. REFERENCE INTERVALS:

	**0-1 day**		**2 days – 90 days**		**91 days – 1 yr**		**2-12 yrs**		**Female 13-99yrs**		**Male 13-99yrs**	
	**L**	**H**	**L**	**H**	**L**	**H**	**L**	**H**	**L**	**H**	**L**	**H**
**RBC (10^12/L)**	4.6	6	3	5.4	3.7	5.3	2.9	5.3	4.1	5.4	4.5	5.8
**HGB (g/dL)**	12.5	19.5	10	14.1	10.5	13.5	11.5	15.5	11	14.5	11.5	15.5
**HCT (L/L)**	0.44	0.64	0.31	0.55	0.33	0.39	0.35	0.45	0.34	0.43	0.35	0.45
**MCV (fL)**	98	118	76	96	70	86	73	95	78	95	78	96
**MCH (pg)**	31	37	28	36	23	31	24	33	26	33	26	33
**MCHC (g/L)**	30	36	31	35	31	35	31	35	31	35	31	35
**RDW**	11.5-16.5											
**WBC (10^9/L)**	6	22	6	20	6	17.5	4.5	14.5	2.4	9.5	2.2	10
**NEUT # (10^9/L)**	4.5	12	1	8.5	1.5	8.5	1.4	9	1	4.8	1	5
**LYMPH # (10^9/L)**	1	6	4	13.5	4	10.5	1.9	9.8	1.2	3.8	1.2	4

**MONO # (10^9/L)**	0.2	1.6	0.2	1.6	0.2	1.3	0.1	1	0.2	0.5	0.2	0.6
**EOS # (10^9/L)**	(0-90 d) 0.1-0.6			(91 d – 13yrs) 0.1-0.8				(14-99yrs) 0.1-0.5				
**BASO # (10^9/L)**	0-0.2											
**NRBC # (10^9/L)**	(0 day) 0-1				(1 day) 0-0.5				(2-5 days) 0-0.1			
**PLT (10^9/L)**	(Female 0-99 yrs) 150-450						(Male 0-99 yrs) 140-400					
**MPV (fl)**	(Female 0-99 yrs) 7-10.5						(Male 0-99 yrs) 7.2-10.5					
**RETICS (10^9/L)**	(0-14 days) 97-385						(15 days-99 yrs) 20-150					
**RETICS %**	(0-14 days) 2-7						(15 days-99 yrs) 0.2-2					

Reference ranges for Omani population adopted from SQUH, 1998 approved by MOH

16. **REFERENCES**:

Abbott Laboratories CD Sapphire Operating Manual.

17. **APPENDICES**:

Not attached to this document.
